# Development of sustained-release extemporaneous moxifloxacin loaded commercial soft hydrogel contact lenses

**DOI:** 10.1016/j.heliyon.2025.e42436

**Published:** 2025-02-01

**Authors:** Duy Toan Pham, Pratthana Chomchalao, Kunasin Bunneramit, Phurichaya Kladcharoen, Rossukon Khotcharrat, Waree Tiyaboonchai

**Affiliations:** a Department of Health Sciences, College of Natural Sciences, Can Tho University, Can Tho, 900000, Viet Nam; b College of Medicine and Public Health, Ubon Ratchathani University, Ubon Ratchathani, 34190, Thailand; c Faculty of Pharmaceutical Sciences, Naresuan University, Phitsanulok, 65000, Thailand; d Faculty of Medicine, Naresuan University, Phitsanulok, 65000, Thailand

**Keywords:** Extemporaneous, Moxifloxacin, Contact lenses, Eye infections, Higuchi, Isotherms

## Abstract

Eye infections such as *Acanthamoeba* keratitis and bacterial keratitis are serious diseases that could lead to severe, sight-threatening complications. Although moxifloxacin eye drops (0.5 % w/v) is accepted for clinical treatments of these infections, the frequent administration is challenging to achieve the adequate dose due to the limitations of low ocular bioavailability and short retention time. To circumvent these issues, this study developed the extemporaneous moxifloxacin loaded commercial soft hydrogel contact lenses with sustained-release property. The simple soaking method was employed on five common available contact lenses of Acuvue, Biomedics, Maxim, Soflens, and Biotrue, which were immersed in the standard moxifloxacin eye drops solutions. Amongst them, three contact lenses (Acuvue, Biomedics, and Maxim) showed high drug loading of ∼2 mg and adequate controllable drug release for 24 h with Maxim possessing the highest release rate, and maintained the effective drug therapeutic level for at least 12 h. Kinetically, both the moxifloxacin loading and releasing processes followed the Higuchi model, with the diffusion mechanism governing the drug behaviors. Isothermally, the moxifloxacin molecules were adsorbed onto the contact lenses surfaces via physical adsorptions by weak interactions of van der Waals forces, ionic bonding, and hydrophobic interactions. Furthermore, both the eye drops brands (Moximac and Zomoxin), the loading pH (6.7 and 6.0), and the loading time (24 h and 2 h) had no significant effects on the loading and release of moxifloxacin, indicating the system versatility. Conclusively, the extemporaneous moxifloxacin loaded contact lenses, with a duration of action of at least 12 h, could be further explored to become a potential treatment for eye infections.

## 1 Introduction

Eye infections such as *Acanthamoeba* keratitis and bacterial keratitis are serious diseases that could lead to severe, sight-threatening complications if not promptly and properly treated [[1], [2], [3], [4]]. *Acanthamoeba* keratitis is caused by a free-living amoeba (*Acanthamoeba* sp.) found in water and soil. It often affects contact lens wearers, especially those who use improper lens care practices, such as using tap water to clean lenses or swimming while wearing lenses. On the other hand, bacterial keratitis is usually caused by bacteria such as *Staphylococcus aureus* or *Pseudomonas aeruginosa*. For these infections, treatment usually involves a combination of antimicrobial eye drops, but the infections are often resistant to treatment, making it a prolonged and challenging process.

Moxifloxacin is a broad-spectrum fourth-generation fluoroquinolone that is generally used for the treatment of eye infections [5,6]. Moxifloxacin is initially approved by the US Food and Drug Administration (FDA) for bacterial conjunctivitis in 2004, then being clinically applied as prophylactic intracameral injection for post-operative endophthalmitis in several literatures [[7], [8], [9], [10], [11]], and recently utilized for bacterial infections prevention after eye surgeries [12,13]. Moreover, in cases of eye drops, moxifloxacin is commonly formulated as 0.5 % w/v solutions (brands Moximac and Zomoxin), which are advised to be administered every 1–2 h as monotherapy regimen for bacterial keratitis [4]. Although possessing efficacy in bacterial endophthalmitis treatments, moxifloxacin eye drops have numerous limitations, namely inadequate drug absorption (biological barriers such as the corneal epithelium and conjunctival absorption can impede drug absorption), low bioavailability of only 1–7%, and high drug loss (due to lacrimal drainage system and eye blinking) [[14], [15], [16]]. These drawbacks lead to frequent eye drop administrations in clinical settings, which consequently create inconvenience for patients and can cause side effects from uncontrolled doses [17,18]. Therefore, an ophthalmic drug delivery system that can mitigate drug loss, prolong the ocular contact time, enhance drug bioavailability, and reduce the administration frequency, is necessary.

To that end, contact lenses are a promising candidate that could provide significantly longer contact times (exceeding 30 min) compared to eye drops (1–3 min), resulting in more than 50 % of the drug released from the contact lens being absorbed into the cornea and anterior eye segment [[19], [20], [21], [22], [23]]. Consequently, this approach enables the potential for reduced drug dosage, decreased administration frequency, and minimized potential adverse effects. Amongst numerous kinds of contact lenses, soft hydrogel contact lenses (the main material is poly(2-hydroxyethyl methacrylate) (pHEMA)) are popularly utilized for drug delivery purposes, mainly due to their highly porous structure that could effectively load/adsorb drug molecules and their swelling property that could controllably release the drugs [24].

To load the drug into the contact lenses, several methodologies exist, including the soaking method, the molecular imprinting method, and the supercritical fluid method. Amongst them, the soaking method stands out as a facile, economical, and convenient approach for extemporaneous pharmaceutical preparations [[25], [26], [27], [28]]. The soaking method involves a simple step of soaking contact lenses in a drug solution, and the drug loading is generated by the difference between drug concentrations in the solution and polymer matrix via chemical interactions such as van der Waals forces, ionic interactions, hydrogen bonding, and hydrophobic interactions [29].

Previously, moxifloxacin has been loaded into the contact lenses as extemporaneous pharmaceutical agents [[30], [31], [32], [33], [34]]. Nevertheless, these research still possessed some limitations individually, and thus, a novel approach is necessary. For instance, Zambelli et al., developed the moxifloxacin loaded silicone contact lenses (Air Optix, Night and Day Aqua) using the soaking method. The drug could be prolonged released for 2 days with the maximum release percentage of 10.6 ± 4.1 %, and the lenses could effectively eliminate *Staphylococcus epidermidis* within 20 min post-application. However, the total amount of drug that could be loaded into and released from the contact lenses was low (212 μg/mL) [35]. Similarly, Sharma et al., studied the loading and release of moxifloxacin from soft contact lenses 1-Day Acuvue at various pH conditions (pH 5.0, 5.5, 5.9, 6.5, and 7.2). Moxifloxacin could be loaded with the highest amount at pH 6.5 (∼1.4 mg) and released 79.84 % after 240 min, following the zero order kinetics. However, this work only investigated one kind of contact lenses, and the release profile was still rapid and uncontrollable [30]. Additionally, Phan et al., incubated the contact lenses with moxifloxacin for 24 h, which might not be a realistic time for extemporaneous preparation [31]. Last but not least, Gade et al., prepared an effective moxifloxacin loaded contact lenses for the treatment of ocular infections, yet these lenses were manufactured by a complex solvent casting method, which might not be appropriate for in-patient care [32].

Taken these aforementioned issues into consideration, this study aimed to investigate commercially available soft hydrogel contact lenses (1-Day Acuvue Moist, Biomedics 1 Day, Biotrue ONEday, Soflens, and Maxim) that possess favorable characteristics for moxifloxacin loading and sustained release via the simple soaking method. The study also explored the factors influencing drug loading and release of moxifloxacin in soft contact lenses, as well as the mathematical kinetics and isotherms underlying the drug adsorption and release processes.

## 2 Materials and methods

### 2.1 Materials

The standard moxifloxacin hydrochloride was imported from Sigma-Aldrich RTC, Laramie, USA. Five commercial contact lenses were used in this study, including 1-day Acuvue (Acuvue, weight 2.0 g/piece, Johnson & Johnson Vision Care Ireland UC, Limerick, Ireland), Biomedics 1 day (Biomedics, weight 1.5 g/piece, Cooper Vision Caribbean Corporation, Rochester, USA), Biotrue ONEday (Biotrue, weight 2.0 g/piece, Bausch & Lomb Incorporated, Rochester, USA), Soflens (weight 1.5 g/piece, Bausch & Lomb Incorporated, Rochester, USA), and Maxim soft lens (Maxim, weight 2.0 g/piece, Cooper Vision Caribbean Corporation, Juana Diaz, USA). The lenses were obtained in their original packaging and the properties of the lenses are listed in Table 1. The Moximac (Micro Labs Limited, Bangalore, India) and Zomoxin (Millimed BFS Co., Ltd, Chiang Rai, Thailand) were designed as commercial moxifloxacin eye drops for testing in this study. Other chemicals of ammonium acetate, acetonitrile, sodium chloride, sodium bicarbonate, calcium chloride, hydrochloric acid, and sterile water, were of reagent grades or higher.

**Table 1:** Properties of commercial contact lenses used in the study.

Contact lenses brand	Material	FDA Classification	Diameter (mm)	Curvature (mm)	Water content (%)	Modulus (MPa) [36,37]
**1-Day Acuvue**	Etafilcon A (pHEMA, PVP, MAA)	IV	14.2	9.0	58	0.20^a^
**Biomedics 1 Day**	Ocufilcon D (pHEMA, MAA)	IV	14.2	8.6	55	0.40
**Biotrue ONEday**	Nesofilcon A (pHEMA, NVP)	II	14.2	8.6	78	0.59
**Soflens 1 Day**	Hilafilcon B (pHEMA, NVP, AMA)	II	14.2	8.6	59	0.20^a^
**Maxim soft lens**	Ocufilcon D (pHEMA, MAA)	IV	14.2	8.6	55	N/A

pHEMA: poly(2-hydroxyethyl methacrylate); AMA: allyl methacrylate; NVP: N-vinyl pyrrolidone; MAA: methacrylic acid; PVP: poly(vinyl pyrrolidone); US FDA classification: Group I: Nonionic (<1 % ionized groups at pH 7.2), low water content (<50 %); Group II: Nonionic (<1 % ionized groups at pH 7.2), high water content (>50 %); Group III: Ionic (>1 % ionized groups at pH 7.2), low water content (<50 %); Group IV: ionic (>1 % ionized groups at pH 7.2), high water content (>50 %); and Group V: Silicone hydrogel lenses; a: tensile modulus based on MCZT (measured central zone thickness).

### 2.2 Measurement of moxifloxacin loading and release amount in contact lenses

High performance liquid chromatography (HPLC) method was used to quantify moxifloxacin amounts in all experiments. To construct the calibration curve, the standard moxifloxacin was dissolved and diluted in mobile phase (ammonium acetate buffer pH 4 and acetonitrile at a ratio of 70:30 v/v) to get the concentration range of 1, 2.5, 5, 10, 20, and 40 μg/mL. These solutions were subjected to HPLC system (Shimadzu, Japan) with the ACE Generix C18 column (150 mm × 4.6 mm, 5 μm), UV detector wavelength of 294 nm and a flow rate of 0.8 mL/min at 25°C [38]. The calibration curve was generated (y = 154556x + 10608, R^2^ = 0.9999) for quantifying the amount of moxifloxacin. The samples were diluted in the mobile phase and analyzed using HPLC with similar conditions to the standard solution and the amount of moxifloxacin was quantified using the calibration curve.

### 2.3 Experimental setup and preliminary tests for the moxifloxacin loading and release in contact lenses

To find the optimal contact lenses for the extemporaneous delivery of commercial moxifloxacin, the standard moxifloxacin was loaded into 5 commercial contact lenses (Acuvue, Biomedics, Biotrue, Soflens, and Maxim), followed by the drug release evaluations.

• **Drug loading study**

For the loading process, the contact lenses were initially soaked in water for 5 min. Then, the contact lenses were immersed in 3 mL of moxifloxacin standard solution (0.5 % w/v, pH 6.7), for 24 h at room temperature. At each specific time point of 0.25, 0.5, 1, 2, 5, 8, and 24 h, the drug solution was withdrawn to determine the moxifloxacin amount by HPLC method. Then, the drug loading amount in the contact lenses was calculated by Equation (1).

(1) Drug loading amount (mg) = Initial drug amount – Drug amount at time point t

• **Drug release study**

**Fig. 1:**
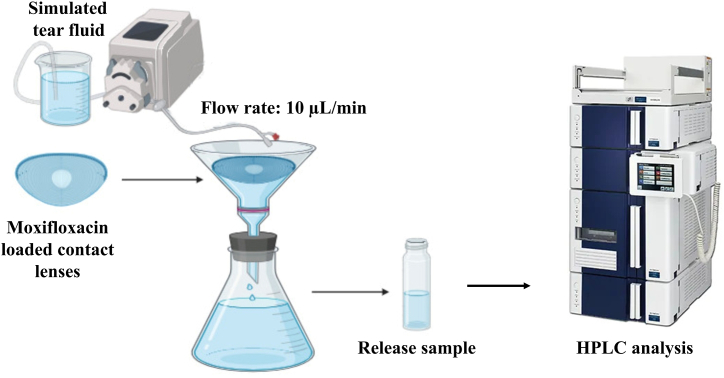
Experimental setup for the *in vitro* moxifloxacin release from the contact lenses.

After the drug loading process, the moxifloxacin loaded contact lenses were partially dried on lens paper and evaluated for their release behaviors at room temperature. To simulate the ocular condition, the lenses were placed in a funnel and the simulated tear fluid (STF, composed of 0.67 g NaCl, 0.2 g NaHCO_3_, 0.008 g CaCl_2_, and 99.122 g, adjusted pH to 7.4) was gradually dropped onto the contact lenses with a flow rate of 10 μL/min using a peristaltic pump (Fig. 1). The experiment was conducted for a period of 24 h, with samples taken at 1, 2, 3, 5, 8, 12, and 24 h. The drug release amount was measured using HPLC and the cumulative drug release (%) over time (h) was plotted [39].

### 2.4 Extemporaneous moxifloxacin loaded contact lenses

After experimental setup and preliminary tests with the standard moxifloxacin solution, three optimal contact lenses (Acuvue, Biomedics, and Maxim) were selected for the extemporaneous moxifloxacin loaded contact lenses. For this, the drug-loaded contact lenses were manufactured similarly to the process described in section 2.3. To mimic the actual clinical situation, the contact lenses were immersed in the commercial moxifloxacin eye drop, Moximac (0.5 % w/v, pH 6.7), instead of using the moxifloxacin standard solution. The drug release tests of three drug-loaded contact lenses were performed similarly to those detailed in section 2.3.

To further evaluate the effects of formulation factors on the moxifloxacin loaded contact lenses, the Maxim contact lenses were used as a representative. Three factors were varied, including (1) the eye drop brands, Moximac and Zomoxin, (2) the loading pH, the inherent eye drop pH of 6.7 and 6.0, and (3) the loading time, 2 h and 24 h. The moxifloxacin loading amount and release profiles were the comparison values.

### 2.5 Drug adsorption and release isotherm and kinetics

Various common models were utilized to determine the moxifloxacin loading and release behaviors into/from the contact lenses. These models have been widely utilized for drug adsorption and release characteristics. These included Langmuir model and Dubinin-Radushkevich model for isothermal properties [40]; and zero-order model, first-order model, and the Higuchi model for drug adsorption and release properties [41]. All the fitting raw data and fitting graphs are included in the Supporting Information (**Supplementary file**).

For the loading/adsorption process, the standard Langmuir model (Equation (2)) and Dubinin-Radushkevich model (Equation (3)) were used to evaluate the isothermal property, whereas the zero-order model (Equation (4)), the first-order model (Equation (5)), and the Higuchi model (Equation (6)) were used to elucidate the adsorption kinetics.

(2) $$\frac{q_{e}}{q_{m}}=\frac{K_{L}C_{e}}{1+K_{L}C_{e}}$$

(3) $$\ln\;q_{e}=\ln\;q_{m}-\beta \varepsilon^{2}$$

(4) $$q_{t}=q_{e}+k_{0}t$$

(5) $$\ln\left(q_{e}\;–\;q_{t}\right)=\ln\left(q_{e}\right)\;–\;k_{1}t$$

(6) $$q_{t}=k_{H}t^{1/2}$$

where C_e_ is the moxifloxacin equilibrium concentration (mg/L), q_e_, q_m,_ q_t_ are the adsorption capacities at equilibrium, at maximum values, and at time point t (mg/g), respectively, K_L_, β, k_0_, k_1_, and k_H_ are the Langmuir constant (L/mg), the Dubinin-Radushkevich constant (mol^2^/kJ^2^), the zero-order constant, the first-order constant, and the Higuchi constant, respectively, and $\varepsilon =\text{RTln}\left(1+\frac{1}{\text{Ce}}\right)$ is the Polanyi potential energy.

Due to the fact that the moxifloxacin loading and releasing processes from/to the contact lenses were similar, both processes were fitted utilizing the same mathematical models to compare both adsorption/release behaviors. Therefore, the zero-order model (Equation (4)), the first-order model (Equation (5)), and the Higuchi model (Equation (6)) were utilized to evaluate the release kinetics, which are similar approaches to those of the loading process. In these cases, q_t_ is the cumulative amount of moxifloxacin release at time point t.

### 2.6 Calculation of therapeutic level

In a clinical setting, the doses of general moxifloxacin eye drops (0.5 % w/v) for treating bacterial keratitis is 1 drop (∼50 μL) every 2 h, 12 times/day [4]. Thus, the amount of moxifloxacin that the patient used daily is 3 mg. However, the ocular bioavailability of the eye drops is only ∼5 %, making the therapeutic level of moxifloxacin become 150 μg/day. On the other hand, the ocular bioavailability of contact lenses is at least ∼50 % [42]. Therefore, the therapeutic requirement of moxifloxacin from contact lenses is 300 μg/day. In other words, the contact lenses should release moxifloxacin for at least 12.5 μg/h to maintain the drug therapeutic level.

### 2.7 Statistical analysis

All quantitative experiments were conducted in triplicate, except for the preliminary tests (section 2.3), which was conducted once. Experimental results are reported as mean ± standard deviation (SD). Statistical analyses were conducted using the paired *t*-test or one-way ANOVA with Bonferroni post-hoc test, with p < 0.05 for significant differences.

## 3 Results and discussions

Moxifloxacin is a fluoroquinolone bactericidal drug that is commonly used in treating bacterial endophthalmitis and preventing bacterial infection after eye surgery. However, the clinical approach of using moxifloxacin eye drops (0.5 % w/v, common brands of Moximac and Zomoxin) possesses numerous limitations of poor ocular absorption, low bioavailability (∼5 %), and frequent administration (i.e., administer every 2 h). Therefore, to circumvent this issue, the extemporaneous moxifloxacin loaded contact lenses show much potential by prolonging the ocular residence time, reducing drug loss, and controlling the drug release. All the experimental raw data are included in the Supporting Information (**Supplementary file**).

### 3.1 Experimental setup and preliminary tests on the moxifloxacin loading and release in contact lenses

**Fig. 2:**
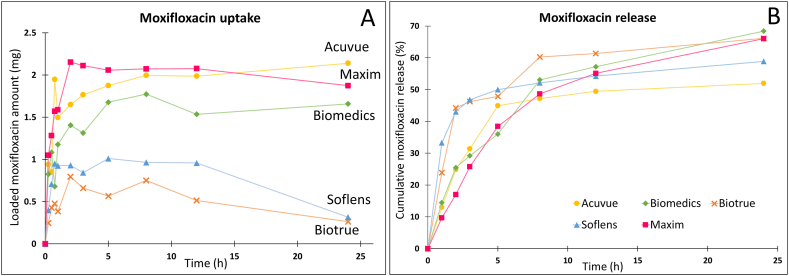
(A) Amount of moxifloxacin loading and (B) moxifloxacin release profiles of 5 commercial contact lenses (Acuvue, Maxim, Biomedics, Soflens, and Biotrue) (n = 1).

To that end, this study first investigated the ability to load and release the moxifloxacin into/from five commercial contact lenses (Acuvue, Biomedics, Maxim, Soflens, and Biotrue) (Fig. 2). Using the simple soaking method, the maximum amount of loaded moxifloxacin followed the order of Maxim (2.15 mg) > Acuvue (2.14 mg) > Biomedics (1.77 mg) > Soflens (1.01 mg) > Biotrue (0.79 mg) (Fig. 2A). Similarly, the cumulative moxifloxacin release percentages at 24 h of Maxim, Acuvue, Biomedics, Soflens, and Biotrue contact lenses were 66.01 %, 51.98 %, 68.41 %, 58.91 %, and 66.13 %, respectively (Fig. 2B). It is worth to notice that there were small fluctuations in the moxifloxacin uptake/adsorption process, which could be due to the nature dynamic of the adsorption/desorption/resorption processes, or the measurement artifacts. Adsorption is the process where the drug molecules from a gas, liquid, or dissolved solid adhere to the surface of a solid or liquid (the adsorbent). This process can be physical (physisorption) or chemical (chemisorption) depending on the nature of the interactions. Whereas, desorption is the reverse process of adsorption, where the adsorbed molecules or particles detach from the surface and return to the surrounding phase [43]. Since the adsorption of the moxifloxacin on the contact lenses is a dynamic process, the drug molecules bind/adsorb to the lenses materials via weak physical interactions (i.e., van der Waals forces, ionic interactions, hydrogen bonding, and hydrophobic interactions), discussed in the next sections, and, at the same time, unbind/desorb from the lenses materials due to competitive interactions with the solvent molecules. The unavoidable thermal Brownian motion of the molecules causes random adsorption and desorption events [44], leading to fluctuations in the adsorbed amount of moxifloxacin on the contact lenses surfaces. Factors affecting this fluctuation included (1) lens material, in which hydrogel lenses (the lenses used in this study) are highly water-absorbing, allowing for significant drug loading initially due to their hydrophilic nature, but the material's equilibrium with the surrounding medium may lead to some back-diffusion of the drug into the solution, causing fluctuations [45]; (2) lens shrinking, in which the lens matrix might slightly shrink or re-equilibrate with its environment after reaching peak saturation (due to its high water content and network flexibility), leading to a re-release of moxifloxacin into the solution; and (3) lens pore structure and diffusivity, in which the hydrogel lenses possess lots of pores and the larger pore sizes allow for greater drug diffusion into the lens but also permit easier release, contributing to non-linear uptake curves [46].

**Fig. 3:**
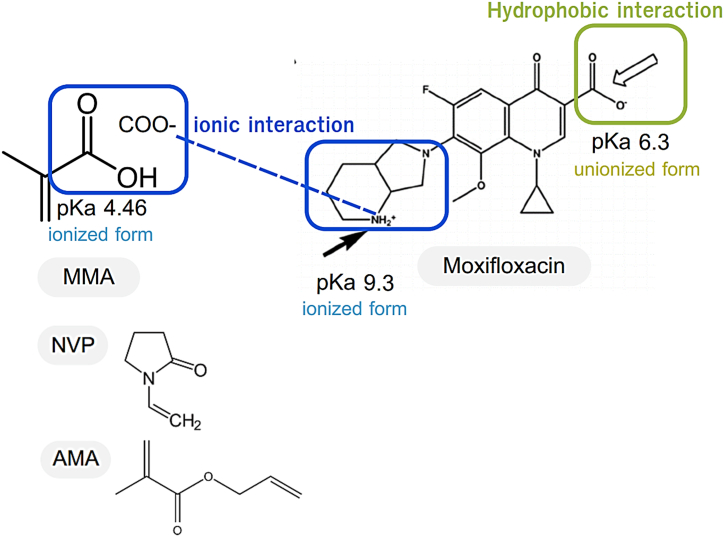
Schematic interactions between the drug moxifloxacin and the contact lenses materials during the drug loading process at pH 6.7.

The fact that three contact lens brands, Acuvue, Biomedics, and Maxim, loaded more amount of moxifloxacin than the other two, Soflens, and Biotrue, could be due to the lens's materials. The Acuvue, Biomedics, and Maxim contain methacrylic acid (MAA) (Table 1), which has a carboxylic acid group in its structure with a pKa of 4.46 [30]. Thus, when immersed in the moxifloxacin solution at pH 6.7, the carboxylic acid group of MAA was in an ionized form, which consequently ionic interacted with the moxifloxacin amine group (pKa = 9.3) [47] (Fig. 3). This ionic interaction provided more moxifloxacin adsorption on the surfaces of these contact lenses. On the other hand, Soflens and Biotrue do not have MAA as a lens's materials, but contain allyl methacrylate (AMA) and N-vinyl pyrrolidone (NVP), which are non-ionized molecules at pH 6.7. Nevertheless, Soflens and Biotrue contact lenses can still adsorb some moxifloxacin molecules, possibly via the hydrophobic interactions and hydrogen bonding between the drug and the contact lenses materials. Our findings are supported by existing literature that drug loading and release control are influenced by the affinity of interactions between the drug and polymer composed in the lens for contact lenses-based drug delivery systems. For example, Topete A. et al., investigated drug loading condition for the drug-loaded ophthalmic lens to improve sustained drug delivery. Moxifloxacin, diclofenac, and ketorolac were tested in this study and the possible interactions between the drug molecules and polymeric matrix of intraocular lens was examined using ssNMR spectroscopy. The results have shown that hydrogen bonding and ionic interactions associated with the interactions between chemical groups present in the main components of polymeric materials (HEMA and MMA) and moxifloxacin [48].

In summary, three contact lenses of Acuvue, Biomedics, and Maxim, were selected for the extemporaneous moxifloxacin loading and release studies.

### 3.2 Extemporaneous moxifloxacin loaded contact lenses

**Fig. 4:**
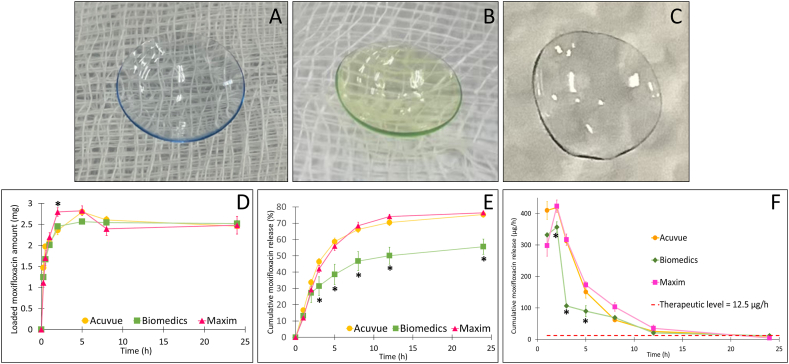
(A–C) The contact lenses images; (A) the unloaded lens, (B) the moxifloxacin loaded lens, and (C) the moxifloxacin loaded lens after the release test for 24 h. (D) The amount of moxifloxacin loaded in the contact lenses, (E) the cumulative release of moxifloxacin from the contact lenses, and (F) the moxifloxacin release amount in relation with the therapeutic level (n = 3). ∗ denotes significant differences (p < 0.05) between samples at the same timepoint.

Physically, the contact lenses before (Fig. 4A) and after the moxifloxacin loading process for 24 h (Fig. 4B) demonstrated no significant differences in shape, with the main difference being the color (i.e., the moxifloxacin has a green-yellowish color). Moreover, after conducting the drug release test for 24 h, the contact lenses reverted to their original color (Fig. 4C), suggesting most of the drug was released during that period. Additionally, the transparency test was conducted by measuring the lenses %transmittance using UV–Vis spectroscopy at 280–315 nm (UV B range), 316–380 nm (UV A range), and 381–780 nm (visible range), following the standard protocol [49]. The results showed that all the lenses, both before the moxifloxacin loading, after the moxifloxacin loading, and after moxifloxacin release, possess a transmittance of >90 %, indicating the appropriate optical clarity [50].

Quantitatively, all three contact lenses could adsorb moxifloxacin rapidly, reaching maximum capacity at 5 h (Fig. 4D), with the amount of 2.80 ± 0.05 mg, 2.57 ± 0.05 mg, and 2.83 ± 0.11 mg, for Acuvue, Biomedics, and Maxim, respectively. Additionally, the moxifloxacin was gradually released from the contact lenses for the entire testing duration of 24 h, reaching its peaks at 75.32 ± 1.77 %, 55.62 ± 4.66 %, and 76.39 ± 1.05 %, for Acuvue, Biomedics, and Maxim, correspondingly (Fig. 4E). In terms of the therapeutic level, as mentioned in section 2.6, the minimum released moxifloxacin amount from the contact lenses should be 12.5 μg/h to maintain the antibacterial action. Thus, the cumulative release percentages of moxifloxacin were re-calculated regarding the release rate (Fig. 4F). The results showed that all three contact lenses could sustain the moxifloxacin amount above the therapeutic level for at least 12 h, which was superior to the conventional eye drops that need frequent administrations of every 2 h.

Kinetically, all three contact lenses followed well with the Higuchi model, for both the moxifloxacin loading and release processes, indicating that the drug adsorption and releasing were governed by the same mechanisms (Table 2). The drug loading rates of the contact lenses followed the order of Maxim > Acuvue = Biomedics, whereas the drug release rates followed Maxim = Acuvue > Biomedics. The Higuchi model is used to determine the drug release rate from matrix platforms, in which the loading drug amount exceeds the solubility in the matrix medium [51]. Thus, it was confirmed that the moxifloxacin released from the contact lenses mainly via the diffusion mechanism [52]. This fact benefits most of the topical delivery systems, including the present contact lenses, since the drug release could be favorably controlled by the concentration gradients, consequently leading to an adequate initial drug amount and a sustained drug release afterward.

**Table 2:** Moxifloxacin loading/adsorption and release kinetics (zero order, first order, and Higuchi model) R^2^ values of three different drug loaded contact lenses of Acuvue, Biomedics, and Maxim.

Contact lenses brand	Coefficient of determination (R^2^)					
	Moxifloxacin loading process			Moxifloxacin release process		
	Zero order	First order	Higuchi	Zero order	First order	Higuchi
**Acuvue**	0.5711	0.6763	**0.8510 (k**_**H**_**= 1.6279)**	0.8642	0.5312	**0.9696 (k**_**H**_**= 25.2970)**
**Biomedics**	0.7291	0.8615	**0.9504 (k**_**H**_**= 1.7206)**	0.8545	0.5240	**0.9791 (k**_**H**_**= 17.1536)∗∗**
**Maxim**	0.8179	0.8757	**0.9844 (k**_**H**_**= 2.0026)∗**	0.9234	0.5960	**0.9635 (k**_**H**_**= 25.9360)**

**Bold:** the most fitted models; ∗, ∗∗: significant differences compared with the other lenses.

Isothermally, the contact lenses were fitted with the Langmuir and Dubinin-Radushkevich model (Table 3). Langmuir and Dubinin-Radushkevich are two of the most common models utilized to understand the adsorption behaviors [53]. The Langmuir model explains monolayer adsorption based on the assumptions that molecules are adsorbed onto fixed sites, all of which have the same energy, and that there is no interaction between the adsorbed molecules and the surrounding sites [54,55]. On the other hand, the Dubinin-Radushkevich model describes the adsorption process on adsorbents that have a pore structure or a heterogeneous surface, which is based on the filling of micropore volume during the adsorption process [56]. This model also estimates the mean free energy of adsorption, which can indicate whether the process is physical or chemical adsorption [57]. The results demonstrated that all three lenses followed well with the Dubinin-Radushkevich model, rather than the Langmuir model, with the R^2^ of the Dubinin-Radushkevich model of >0.95 (Table 3). This suggested that the moxifloxacin adsorption onto the contact lenses followed the pore filling/adsorption process on a heterogeneous surface.

**Table 3:** Moxifloxacin loading/adsorption isotherms (Langmuir model and Dubinin-Radushkevich (D-R) model) mathematical values of three different drug loaded contact lenses of Acuvue, Biomedics, and Maxim.

Contact lenses brand	Langmuir model			D-R model		
	R^2^	q_m_ (mg/g)	q_e_ (mg/g)	R^2^	β	E (kJ/mol)
**Acuvue**	0.9556	0.12	1.11	**0.9765**	171.65	0.076
**Biomedics**	0.9593	0.08	1.03	**0.9824**	192.53	0.072
**Maxim**	0.9236	0.09	1.13	**0.9568**	183.40	0.074

R^2^: coefficient of determination; q_m_: theoretical maximum adsorption capacity; q_e_: experimental adsorption capacity; β: D-R constant; E: adsorption energy.

Furthermore, from the Dubinin-Radushkevich model, the adsorption energy (E, kJ/mol) could be calculated as $E=\frac{1}{\sqrt{2\beta }}$. An E value of less than 8 kJ/mol suggests that the adsorption process is mainly a physical-driven process, while the E values of 8–16 kJ/mol indicate the chemical-driven process. Since all contact lenses show relatively low E values (Table 3), it could be concluded that the moxifloxacin loading/adsorption process happened mainly via physical interactions (i.e., van der Waals forces, hydrogen bonding, ionic interactions, and hydrophobic interactions). This fact was in agreement with the discussion in section 3.1, regarding the potential interactions between moxifloxacin and the contact lenses materials. Moreover, these physical interactions might be unstable, and thus, easily broken (i.e., desorption) and released the drug molecules back into the media, making slight decreases in the adsorption curves (Fig. 4D). It is also worth to note that the E value of Biomedics was the lowest amongst three lenses, thus theoretically, it should have the least binding between the contact lenses and the drug, making the drug rapidly releases. Contradictory, the opposite phenomenon was observed. Therefore, the release process might not be governed by this factor alone. Conclusively, both the Langmuir and Dubinin-Radushkevich model might not be the only models that describe the adsorption behaviors of the contact lenses, further research are necessary to elucidate this issue.

### 3.3 Drug loading factor variations

Since Maxim possessed the highest drug loading and release rates, this contact lens was selected for the experiments on the drug loading factor variations.

**Fig. 5:**
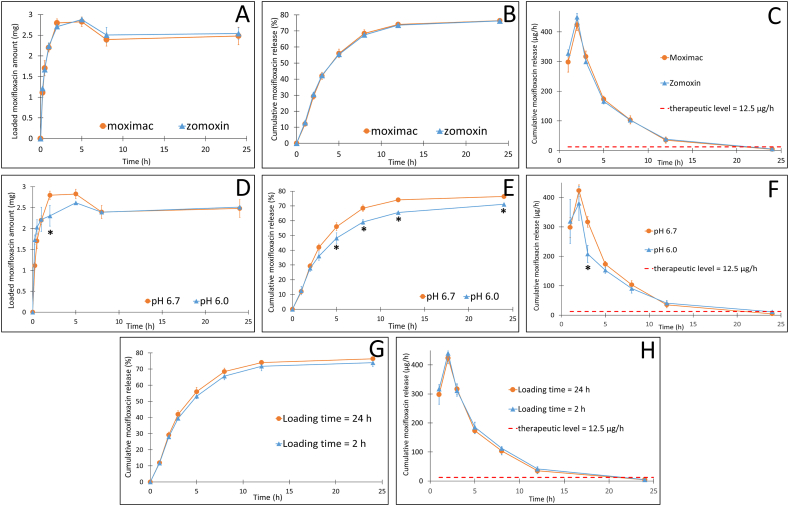
(A and D) The moxifloxacin loading process into the contact lenses Maxim, at different (A) eye drops (Moximac and Zomoxin) and (D) loading pH (6.7 and 6.0). (B, E, and G) The drug release process from the moxifloxacin loaded Maxim contact lenses at varied factors. (C, F, and H) the drug release amount in relation with the therapeutic level (n = 3). ∗ denotes significant differences (p < 0.05) between samples at the same timepoint.

Regarding the eye drops brands, Moximac and Zomoxin, both eye drops could be loaded effectively into the contact lenses, as well as released controllably from the lenses, with no significant differences in all investigated parameters. Specifically, the maximum amount of moxifloxacin that could be loaded in the contact lenses was 2.83 ± 0.11 mg and 2.88 ± 0.02 mg, for Moximac and Zomoxin, respectively (Fig. 5A). The percentages of moxifloxacin released from the contact lens after 24 h was 76.39 ± 1.05 % and 76.23 ± 1.01 %, for Moximac and Zomoxin, respectively (Fig. 5B), with both followed the Higuchi model (Table 4). The adsorption process for both eye drops also similarly followed the Langmuir and Dubinin-Radushkevich model (Table 5). Therefore, the brand of eye drops did not affect the moxifloxacin loading process onto the lenses.

**Table 4:** Moxifloxacin loading/adsorption and release kinetics (zero order, first order, and Higuchi model) R^2^ values of the Maxim contact lenses, at different varied factors of the eye drop brands, the loading pH, and the loading time.

Varied factor	Coefficient of determination (R^2^)					
	Moxifloxacin loading process			Moxifloxacin release process		
	Zero order	First order	Higuchi	Zero order	First order	Higuchi
**Eye drop brand (Moximac and Zomoxin)**						
Moximac	0.8179	0.8757	**0.9844 (k**_**H**_**= 2.0026)**	0.9234	0.5960	**0.9635 (k**_**H**_**= 25.9360)**
Zomoxin	0.7849	0.8721	**0.9736 (k**_**H**_**= 1.9387)**	0.9137	0.5798	**0.9683 (k**_**H**_**= 25.4920)**
**Loading pH (6.7 and 6.0)**						
pH 6.7	0.8179	0.8757	**0.9844 (k**_**H**_**= 2.0026)**	0.9234	0.5960	**0.9635 (k**_**H**_**= 25.9360)**
pH 6.0	0.4580	0.5167	**0.7581 (k**_**H**_**= 1.5779)**	0.9150	0.5758	**0.9753 (k**_**H**_**= 22.0543)∗**
**Loading time (24** **h and 2** **h)**						
24 h	0.8179	0.8757	**0.9844 (k**_**H**_**= 2.0026)**	0.9234	0.5960	**0.9635 (k**_**H**_**= 25.9360)**
2 h	N/A	N/A	N/A	0.9283	0.5987	**0.9657 (k**_**H**_**= 24.7516)**

**Bold:** the most fitted models; N/A: not available due to short loading time with inadequate timepoints to fit in the models ∗: significant differences compared with the other variables.

**Table 5:** Moxifloxacin loading/adsorption isotherms (Langmuir model and Dubinin-Radushkevich (D-R) model) mathematical values of the Maxim contact lenses, at different varied factors of the eye drop brands and the loading pH.

Varied factor	Langmuir model			D-R model		
	R^2^	q_m_ (mg/g)	q_e_ (mg/g)	R^2^	β	E (kJ/mol)
Eye drop brand (Moximac and Zomoxin)						
Moximac	0.9236	0.09	1.13	0.9568	183.40	0.074
Zomoxin	0.9401	0.10	1.15	0.9681	184.07	0.074
**Loading pH (6.7 and 6.0)**						
pH 6.7	0.9236	0.09	1.13	0.9568	183.40	0.074
pH 6.0	0.9877	0.10	1.05	0.9930	208.86	0.069

R^2^: coefficient of determination; q_m_: theoretical maximum adsorption capacity; q_e_: experimental adsorption capacity; β: D-R constant; E: adsorption energy.

In terms of the drug loading pH, at both pH 6.7 and 6.0, the contact lenses could load similar moxifloxacin amounts (2.62 ± 0.02 mg at pH 6.0 and 2.83 ± 0.11 mg at pH 6.7) (Fig. 5C) and release similar maximum moxifloxacin amount after 24 h (71.15 ± 0.60 % at pH 6.0 and 76.39 ± 1.05 % at pH 6.7), which followed the Higuchi model (Fig. 5D). Furthermore, contact lenses fabricated at both pH followed the Langmuir and Dubinin-Radushkevich model (Table 5). Interestingly, the release rate of moxifloxacin from contact lenses loaded at pH 6.0 was significantly lower than that at pH 6.7 (p = 0.0225) (Table 4). The E value of contact lenses fabricated at pH 6.0 was lower than that at pH 6.7, the lower the binding energy, the weaker the bonding forces between moxifloxacin and the contact lens's materials, consequently resulting in faster release rates. Ironically, the opposite phenomenon was observed, once again, suggesting that the release process might be more complex, which needs further in-depth research. Nevertheless, both lenses could maintain the moxifloxacin therapeutic levels for 12 h (Fig. 5F). Thus, there is no need to adjust the pH of the moxifloxacin solution used in the lens loading step.

Finally, to shorten the contact lenses soaking time, the moxifloxacin loading time of 2 h was investigated. Compared to the contact lenses with loading time of 24 h, the ones with 2-h loading time showed similar parameters of drug loading (2.71 ± 0.02 mg at 2 h and 2.48 ± 0.21 mg at 24 h), drug release (Fig. 5G), and release rate (Fig. 5G and Table 4). Therefore, the duration of soaking contact lenses in moxifloxacin eye drop solution during the drug loading process could be reduced to 2 h, which provides time-benefits in the clinical settings.

## 4 Conclusions

This study successfully prepared extemporaneous moxifloxacin loaded commercial soft hydrogel contact lenses as a convenient system for the treatment of eye infections. The optimal formula takes only 2 h to prepare, can be used with any brand of eye drops, and does not require adjusting the eye drops pH. Moreover, the contact lenses could load a high drug amount of ∼2.8 mg, sustained drug release for 24 h, and maintained its therapeutic level for at least 12 h. The drug adsorption was a physical process and the drug release rate followed the Higuchi model, which could be favorably controlled. In summary, the product could be further explored to become a potential clinical therapeutic option for eye infection treatments that could enhance the moxifloxacin bioavailability and efficacy, and reduce the administration frequency.

## CRediT authorship contribution statement

**Duy Toan Pham:** Writing – review & editing, Writing – original draft, Resources, Conceptualization. **Pratthana Chomchalao:** Writing – review & editing, Methodology. **Kunasin Bunneramit:** Writing – original draft, Resources, Methodology, Investigation. **Phurichaya Kladcharoen:** Writing – original draft, Resources, Methodology, Investigation. **Rossukon Khotcharrat:** Writing – review & editing, Project administration, Conceptualization. **Waree Tiyaboonchai:** Writing – review & editing, Project administration, Conceptualization.

## Ethical approval statement

Not applicable.

## Data availability statement

Data included in article/supp. material/referenced in article.

## Funding

None.

## Declaration of competing interest

The authors declare that they have no known competing financial interests or personal relationships that could have appeared to influence the work reported in this paper.
